# Effects of short‐term exposure to *Pomacea canaliculata* secretions on *Limnodrilus hoffmeisteri* and *Propsilocerus akamusi*: A study based on behavior, intestinal microbiota, and antioxidant system

**DOI:** 10.1002/ece3.11591

**Published:** 2024-06-25

**Authors:** Mingyuan Liu, Changrun Sui, Baolong Wang, Ruipin Huang, Weixiao Zhang, Tao Zhang, Qian Zhang, Ying Liu

**Affiliations:** ^1^ School of Life Science Liaoning Normal University Dalian China; ^2^ Key Laboratory of Environment Controlled Aquaculture (Dalian Ocean University) Ministry of Education Dalian China; ^3^ College of Marine Science and Environment Dalian Ocean University Dalian China; ^4^ College of Biosystems Engineering and Food Science Zhejiang University Hangzhou China

**Keywords:** behavior, bioturbators, intestinal microbiota, oxidative stress, *Pomacea canaliculata*

## Abstract

*Pomacea canaliculata* is one of the most notorious invasive aquatic snail, capable of influencing various aquatic organisms through their secretions. *Limnodrilus hoffmeisteri* and *Propsilocerus akamusi* are the most prevalent and powerful bioturbators in aquatic ecosystems. However, the mechanism of *P. canaliculata*'s secretions affecting bioturbators remains unknown. This study aimed to investigate the effects of *P. canaliculata*'s secretion on *L. hoffmeisteri* and *P. akamusi*. *L. hoffmeisteri* and *P. akamusi* were treated for 24 h with *P. canaliculata* and the native species *Bellamya aeruginosa* secretions at different densities (1 or 20). The migration numbers and aggregation rate of *L. hoffmeisteri* indicated that *P. canaliculata* secretion caused *L. hoffmeisteri* to become alert and migrate away from the nucleus community, resulting in poor population identification, especially at high concentrations. Moreover, the antioxidant enzymatic activity, lipid peroxidation, intestinal microbial diversity, and composition of the two bioturbators were analyzed. Superoxide dismutase (SOD) activity and malondialdehyde (MDA) concentration were elevated following *P. canaliculata* secretion treatment, indicating oxidative damage. Furthermore, the composition and diversity of intestinal microbiota of *L. hoffmeisteri* and *P. akamusi* were changed. The abundance of functional microbiota decreased, and pathogenic bacteria such as *Aeromonas* became dominant in the intestines of both bioturbators. The current research evaluates the effects of *P. canaliculata* secretion on the behavior, oxidative stress, and intestinal microbial composition and diversity of two bioturbators, providing new insights into the assessment of post‐invaded ecosystems.

## Graphical abstract

*Pomacea canaliculata* affected bioturbators' behavior, antioxidant system, and gut microbiota; Secretions changed the intestinal microbiota community structure of bioturbators; *Limnodrilus hoffmeister* is a more sensitive bioturbator to the invasion of *P. canaliculata*.

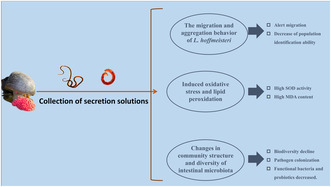

## 1 INTRODUCTION

With economic globalization, invasive species have become a global ecological concern. Due to their formidable adaptability and widespread distribution, invasive species rapidly exploit ecosystem resources, resulting in the decline of native biodiversity and the destruction of community structure and function (Simberloff et al., 2013). These species exhibit diverse ecological strategies and facilitate their population growth through unique ways, such as manipulating the physical and chemical characteristics of the environment (Wright et al., 2012; Zhang, Gu, et al., 2010; Zhang, Hendrix, et al., 2010), as well as the structure or functional groups of habitats (Cui et al., 2011; Hulvey & Zavaleta, 2012). For instance, the invasive crayfish *Procambarus clarkii* has induced changes in the phenotypic traits and developmental timescales of amphibians in the Lombardy region, with complex consequences on their lifetime fitness (Melotto et al., 2020). Furthermore, the invasive herbivorous fish (*Siganus rivulatus*) influences the biological composition of the habitat through excretions and secretions, leading to an increase in phytoplankton biomass and planktonic bacteria abundance (Escalas et al., 2022).

*Pomacea canaliculata*, commonly known as the golden apple snail, is a widespread invasive species (Yang et al., 2023). It has invaded almost all types of freshwater ecosystems since its introduction into China in 1981 and has become a major freshwater invasive species in China. *P. cancaliculata* is able to evade native predators through various adaptive behaviors (Guo et al., 2016). Moreover, *P. canaliculata* has a competitive advantage in the ecosystem due to its strong reproductive capacity and adaptability, and potent shell regeneration ability (Liu et al., 2017; Ueshima & Yusa, 2015). Furthermore, *P. canaliculata* prefers rice and other plant foods (Matsukura et al., 2016), but it can also feed on animals such as *Sinotaia quadrata* and other aquatic animals (Karraker & Dudgeon, 2014; Kwong & Chan, 2009). The feeding behavior of *P. canaliculata* significantly increased the discharge of metabolites and excrement, resulting in nitrogen and phosphorus content elevation, water quality deterioration, and even eutrophication (Wang et al., 2020). The direct predation behavior of *P. canaliculata* and the indirect changes in the aquatic environment will eventually reduce the survival rate of freshwater animals (Maldonado et al., 2019).

Bioturbation is an important ecological process caused by benthic organisms that can alter the physicochemical properties of sediments. Benthos can influence the energy conversion at the sediment–water interface through different activities such as feeding, burrowing, and biological irrigation (Lohrer et al., 2004). The bioturbation ability varies significantly among benthic organisms (Michaud et al., 2005), with Tubificidae being more potent than other benthos (Michaud et al., 2006). Chironomid has a similar high biomass and ability to Tubificidae worms. Tubificidae worms and Chironomid are globally distributed species, considered as the common and influential bioturbators.

*Limnodrilus hoffmeisteri*, one of the most classic tubificidae worms, exhibits the ability to acquire oxygen, construct nests, and feed through swinging movement. Its presence plays a crucial role in the transportation and mixing of sediment particles between deep and shallow layers or between the sediment layer and the overlying water layer, which can affect pH, redox potential, and dissolved oxygen content of the water interface (Zhang, Gu, et al., 2010; Zhang, Hendrix, et al., 2010). *Propsilocerus akamusi*, a widely distributed chironomid species, is a major food source for a variety of aquatic animals in China. *P. akamusi* can also be used to treat water pollution and assess the toxicity of environmental pollutants (Reitzel et al., 2013; Zhang, Gu, et al., 2010; Zhang, Hendrix, et al., 2010).

The invasion of *P. cancaliculata* significantly increases the nitrogen and phosphorus content in the aquatic environment, as well as the food particles and excrement, posing an indirect threat to the native ecosystem and exerting invasive stress on native animals. Previous studies have discovered that Tubificidae and chironomid larvae are abundant species in the ecosystems invaded by *P. cancaliculata* (Maldonado & Martin, 2019). In freshwater ecosystem, *P. akamusi* and *L. hoffmeisteri* often have the same distribution area as *P. cancaliculata*. However, few studies have investigated how invasive stress affects the bioturbation capabilities of Tubificidae and chironomid larvae. Moreover, it remains unknown whether the Tubificidae worms and chironomid larvae can be influenced by the nutrient excretion and chemical cues of *P. cancaliculata*.

In the current study, we investigated the impact of *P. cancaliculata* secretion solutions at varying densities on the migration and aggregation behaviors of *L. hoffmeisteri*, as well as the antioxidant system, and gut microbiota of both *L. hoffmeisteri* and *P. akamusi*. Our findings will provide a theoretical foundation for invasive species assessment and invasion strategies, as well as provide new insights into the management of *P. cancaliculata*.

## 2 MATERIALS AND METHODS

### 2.1 Test organism

*Limnodrilus hoffmeisteri* and *Propsilocerus akamusi* were purchased from a local seafood market (Dalian, China). The invasive species *Pomacea canaliculata* used in the experiment was collected from a stable population in Guangdong Province (Zhaoqing, China). *Bellamya aeruginosa* is a wild population in Weishan Lake obtained from local fishermen and used as the control group (Shandong Province, China). All snails were transported to the laboratory with ice immediately after capture.

*Limnodrilus hoffmeisteri* and *P. akamusi* were temporarily reared in a 3.5 L water tank. Approximately 1000/tank *L. hoffmeisteri* or 100/tank *P. akamusi* were kept separately. The snails *P. canaliculata* and *B. aeruginosa* were separately reared in 76 L aquariums containing 100 animals per species. All species were raised separately at a temperature of 20 ± 2°C and a light/dark (L:D) of 12:12. *L. hoffmeisteri* self‐reproduced for one generation, while *P. akamusi* was stable for 7 days. *P. akamusi* larvae with a body length of 10–14 mm and a dark red color were selected for subsequent experiments. The same diets were used for all the experimental groups to avoid the diet influence on gut microbiota composition. Commercial fish food (Tetramin, Germany) is finely ground into powder and dissolved in distilled water for feeding. Snails and bioturbators are fed at 0.1 and 0.01 mg/ind/day, respectively. Tap water was used for all aquacultures with aeration for at least 48 h.

### 2.2 Preparation of secretion solutions

The secretion solutions were prepared using 1 L of deionized water in a 3.6 L polyethylene container (length 33.7 cm, width 13.3 cm, height 7.5 cm). The high‐density (H) and low‐density (L) solutions were created using either 1 or 20 individuals of *P. canaliculata* (P) with a body mass of 7.00 ± 0.30 g, or *B. aeruginosa* (B) with a body mass of 2.50 ± 0.20 g. These groups were labeled as PL, PH, BL, and BH, respectively. Twenty *P. canaliculata* were chosen as the high‐density group, considering the actual outbreak density of *P. canaliculata* in the freshwater ecosystem is 10 ind/L and the invasion density may continue to elevate. The snails were allowed to live freely in the water for 24 h and then move out. The culture solutions were settled for 1 h to remove the feces, body mucus, and other substances eliminating the potential impacts. The culture solutions were prepared three times and mixed together as the secretion solutions, then frozen at −20°C for the subsequent exposure experiments. The control group (NS group) used aerated tap water as the experimental solution.

### 2.3 Experimental design

In the 6‐well cell culture plate, 600 *L. hoffmeisteri* or 3–4 instar larvae of *P. akamusi* were placed per well, followed by the addition of 15 mL of the prepared secretion solutions from each group. The migration and aggregation behaviors of *L. hoffmeisteri* were observed after 12 h treatment, and each treatment was repeated nine times. The two bioturbators from three wells were used for antioxidant enzyme detection after 24 h treatment and five replicates of intestine contents from five wells were collected for 16S rRNA microbiota sequencing. Test animals and intestine contents were immediately rinsed, frozen in liquid nitrogen, and stored at −80°C for further analysis. All experiments were performed under sterile conditions.

### 2.4 Behavior responses of *L. hoffmeisteri*

*Limnodrilus hoffmeisteri* typically forms large nucleus communities with individuals migrating from the nucleus in response to environmental threats. The migration of *L. hoffmeisteri* from the nucleus can serve as a valuable indicator for evaluating environmental conditions. To evaluate the migratory behavior, 600 *L. hoffmeisteri* were exposed to the secretions of the two snails for 12 h. The proportion of *L. hoffmeisteri* individuals exhibiting migratory behavior was calculated based on the distance of individual worms from the nucleus community or the formation of small sub‐nucleus communities.

To investigate the effects of secretion solutions on the aggregation behavior of *L. hoffmeisteri*, three populations of *L. hoffmeisteri* were arranged in a triangular pattern in the culture plate well, with one population at the top and the other two positioned on the opposite sides at the bottom. Make the three populations form an equilateral triangle. Each population consisted of 100 individuals after 24 h exposure, with the aim of observing their merging into a larger group. The aggregation process typically occurs in three stages. In the first stage, the three small *L. hoffmeisteri* populations extend numerous individuals to touch other populations, thereby identifying them. After identification, the second stage begins, where individuals start pulling each other towards a central point, gradually bringing the three populations closer together. The aggregation behavior of *L. hoffmeisteri* was recorded within 1 h, and the aggregation rate under different treatments was calculated. All the behavioral experiments were repeated nine times.

### 2.5 Antioxidant enzymes detection

After 24 h of acute exposure to different secretion solutions, three *P. akamusi* or 300 *L. hoffmeisteri* were pooled into a 2 mL centrifuge tube and homogenized with an appropriate amount of normal saline. After centrifugation at 2500 rpm/min for 10 min, the supernatant was collected to measure malondialdehyde (MDA), superoxide dismutase (SOD), catalase (CAT), and reduced glutathione (GSH) using commercial assay kits (Nanjing Jiancheng Bioengineering Institute, Nanjing, China). All measurements were standardized by protein concentrations of each sample, which were measured using the bicinchoninic acid (BCA) protein assay kit. All experiments were performed in triplicates.

### 2.6 Intestinal microbiota analysis

After the acute exposure, *L. hoffmeisteri* was washed with sterile physiological saline three times, and 100 worms were mixed into one sample. The intestine of chironomid larvae was quickly separated on ice after rinsing, and the intestine of five larvae was pooled into one sample. All the samples were stored in liquid nitrogen immediately. The total genome DNA was extracted and DNA concentration and purity were monitored. Then the bacterial 16S rRNA gene V4 region was amplified with the primers 515F‐806R (F: CCTAYGGGRBGCASCAG, R: GGACTACNNGGGTATCTAAT). Sequencing was subsequently determined on an Illumina MiSeq platform of Novogene (Beijing, China) and 250 bp paired‐end reads were generated for intestinal microbiota analysis.

### 2.7 Statistical analysis

Data were presented as mean ± standard deviation (SD) and tested for statistical significance using Paired‐sample *t*‐tests. The statistical values were considered significantly different when the calculated probability (*p*) level was below .05.

## 3 RESULTS

### 3.1 Effects of *P. canaliculata* secretion on the migration and aggregation behavior of *L. hoffmeisteri*

**FIGURE 1:**
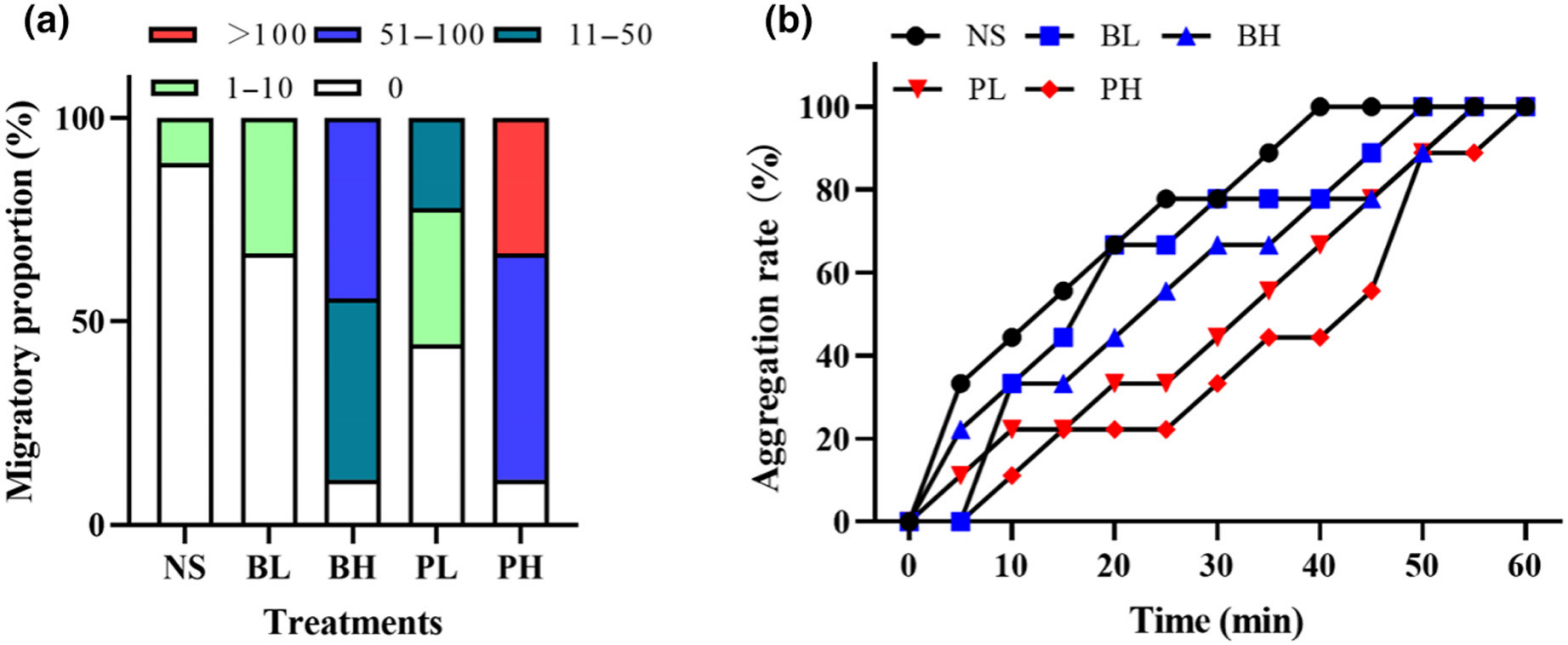
The behavior responses of *Limnodrilus hoffmeisteri* after exposure to secretion solutions (a) migratory proportion of *L. hoffmeisteri*, (b) aggregation rate of *L. hoffmeisteri*. *N* = 9.

The proportions of *L. hoffmeisteri* that migrated from the nuclear population after 12 h of treatment with different secretion solutions varied depending on the type of secretion solutions (Figure 1a). A high proportion of experimental unities in the PH group showed a migratory trend, with over 100 individuals migrating in 33.33% of the unities and 51–100 individuals migrating in 55.56% of the unities. In the BH group, the migration proportion is lower than in the PH group, with only 44.44% of the unities having 51–100 migratory individuals and the rest having less than 50 migratory individuals. The same trends were observed in the low concentration group, with 22.22% of the experimental unities in the PL group having 11–50 migratory individuals, whereas the BL group had no unities with more than 10 migratory individuals, consistent with the NS group.

The aggregation rate of *L. hoffmeisteri* increased over time, with the *P. canaliculata‐*treated group showing significantly lower aggregation than the other groups (*p* < .05). The aggregation rate was higher in the *B. aeruginosa*‐treated group, even at high concentrations. All (100%) *L. hoffmeisteri* in the NS group completely merged into a large population after 40 min, followed by the BL and BH groups (77.78%), the PL group (66.67%), and the PH group (44.44%). Moreover, all *L. hoffmeisteri* populations in the BL group merged after 50 min, 55 min in the PL and BH groups, and 60 min in the PH group (Figure 1b).

### 3.2 Effects of the *P. canaliculata* secretion on the antioxidant system of *L. hoffmeisteri* and *P. akamusi*

**FIGURE 2:**
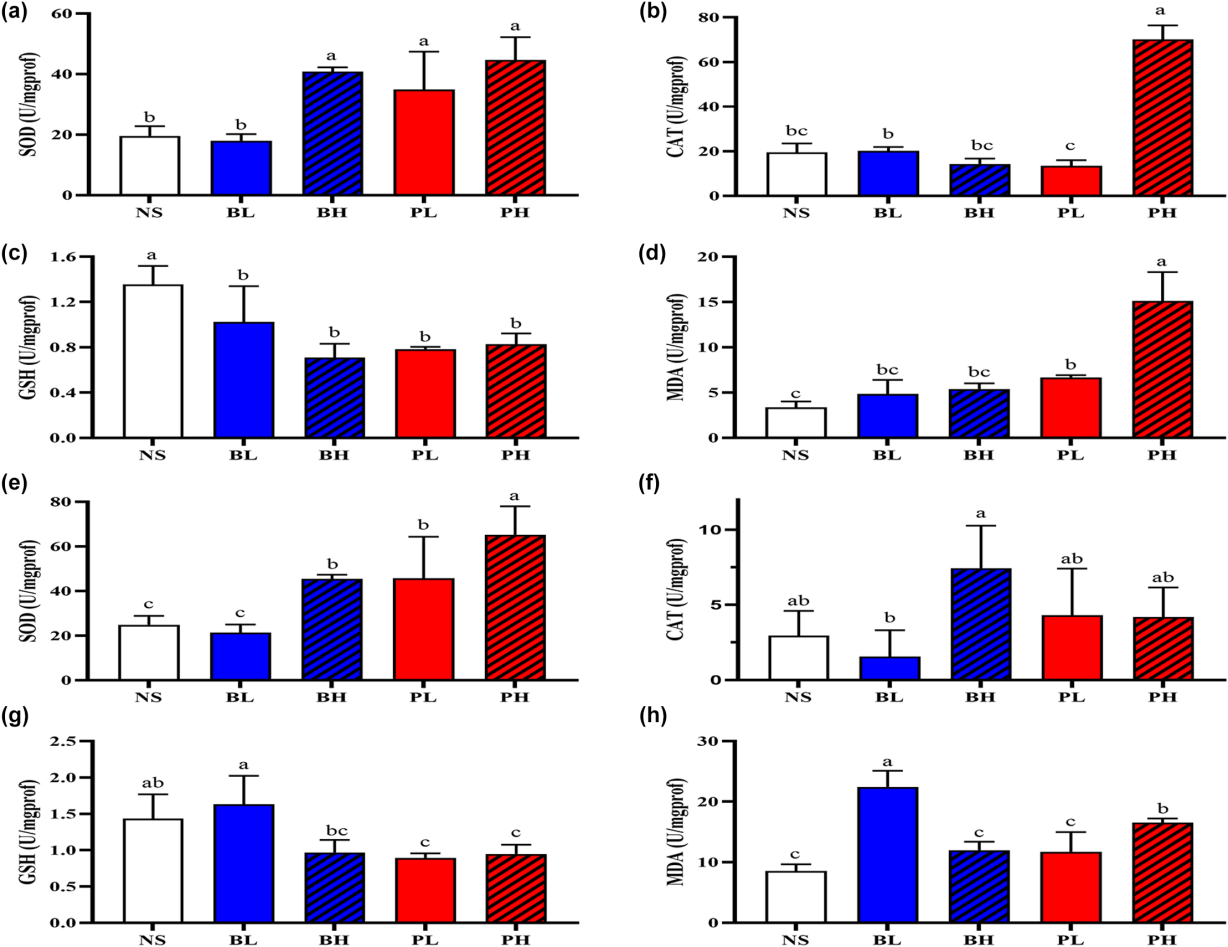
Antioxidant enzymatic activity and lipid peroxidation of *Limnodrilus hoffmeisteri* and *Propsilocerus akamusi* after exposure to secretion solutions. (a–d) represent the activities of SOD, CAT, GSH, and MDA for *L. hoffmeisteri*, (A–D) represent the activities of SOD, CAT, GSH, and MDA for *P. akamusi*. *N* = 3. Different letters indicated significant differences between groups (*p* < .05).

After *L. hoffmeisteri* was treated for 24 h, the SOD activities in the PH, BH, and PL groups were significantly higher than that in the NS group (*p* < .05), whereas the SOD activity in the BL group was equivalent to that in the NS group (Figure 2a). This trend was also observed in *P. akamusi* (Figure 2A). The CAT activity of *L. hoffmeisteri* in the PH group was significantly higher than that in other groups (*p* < .05) (Figure 2b). In contrast, the CAT activity of *P. akamusi* was significantly higher in the BH group than in the BL group (*p* < .05) (Figure 2B). The secretion solutions inhibited the GSH activities of *L. hoffmeisteri* and *P. akamusi*. Compared to the NS group, the *L. hoffmeisteri* population was significantly decreased in the BL, BH, PL, and PH groups (*p* < .05) (Figure 2C), as did the *P. akamusi* population in the PL and PH groups (*p* < .05) (Figure 2C). Both *P. canaliculata* and *B. aeruginosa* secretions increased MDA content in the tested benthic animals. The MDA content increased in a dose‐dependent manner after exposure to *P. canaliculata* secretions, and it was significantly higher in the PH group compared to the PL group for both *L. hoffmeisteri* and *P. akamusi* (*p* < .05) (Figure 2d,D).

### 3.3 Effects of the *P. canaliculata* secretion on the intestinal microbiota of *L. hoffmeisteri* and *P. akamusi*

A total of 832, 1120, 880, 834, and 619 Operational Taxonomic Units (OTUs) were observed in the NS, BL, BH, PL, and PH groups for *L. hoffmeisteri*, respectively. A total of 1235, 869, 1078, 1063, and 428 OTUs were observed in the NS, BL, BH, PL, and PH groups for *P. akamusi*, respectively. The Venn diagram indicated significant alterations in the gut microbiome composition of two bioturbators due to exposure to the secretion solutions of *P. canaliculata* and *B. aeruginosa*. To evaluate the abundance and diversity of microbiota, the Chao1, Shannon, and Simpson indices were used (Table 1). It revealed that exposure to low‐density *B. aeruginosa* secretion increased the diversity of the host intestinal microbiota in *L. hoffmeisteri*. The highest value for the Shannon index was observed in the BH group, while the Chao1 index decreased slightly.

**TABLE 1:** Intestinal microbial diversity of *Limnodrilus hoffmeisteri* and *Propsilocerus akamusi* under different treatments.

Species	Group	OTU	Chao1	Shannon	Simpson
*L. hoffmeisteri*	NS	475.333 ± 86.333^a^	479.787 ± 89.950^a^	4.922 ± 0.430	0.875 ± 0.032
	BL	560.667 ± 56.667^a^	565.498 ± 56.498^a^	5.164 ± 0.338	0.844 ± 0.028
	BH	466.000 ± 53.000^a^	467.814 ± 51.147^a^	5.268 ± 0.851	0.887 ± 0.037
	PL	370.333 ± 211.667^ab^	375.852 ± 214.719^ab^	4.034 ± 1.905	0.726 ± 0.215
	PH	276.333 ± 99.667^b^	286.419 ± 104.837^b^	3.705 ± 1.273	0.769 ± 0.149
*P. akamusi*	NS	631.000 ± 170.000^a^	631.667 ± 171.297^a^	6.160 ± 1.272	0.906 ± 0.072
	BL	359.000 ± 300.000^ab^	361.063 ± 300.080^ab^	3.885 ± 2.624	0.684 ± 0.396
	BH	569.333 ± 89.667^a^	572.668 ± 86.787^a^	5.577 ± 1.219	0.893 ± 0.070
	PL	475.333 ± 233.667^ab^	476.136 ± 233.520^ab^	5.423 ± 1.523	0.887 ± 0.085
	PH	219.000 ± 106.000^b^	219.684 ± 106.191^b^	3.719 ± 1.235	0.801 ± 0.101

*Note*: Different letters indicated significant differences between groups (*p* < .05).

Conversely, the intestinal microbial diversity of *L. hoffmeisteri* decreased after exposure to *P. canaliculata* secretion. The Chao1 index significantly decreased (*p* < .05) after exposure to high‐density *P. canaliculata* secretion. Moreover, the Shannon and Simpson indices of the PH group were lower than those of the PL and NS groups. A similar trend was observed for *P. akamusi*, where exposure to *P. canaliculata* secretion decreased the intestinal microbial diversity of the host; the Chao1 index of the PH group was significantly lower than that of the NS group (*p* < .05), and the values of Shannon and Simpson indices in the treated groups were also lower than that in the NS group.

**FIGURE 3:**
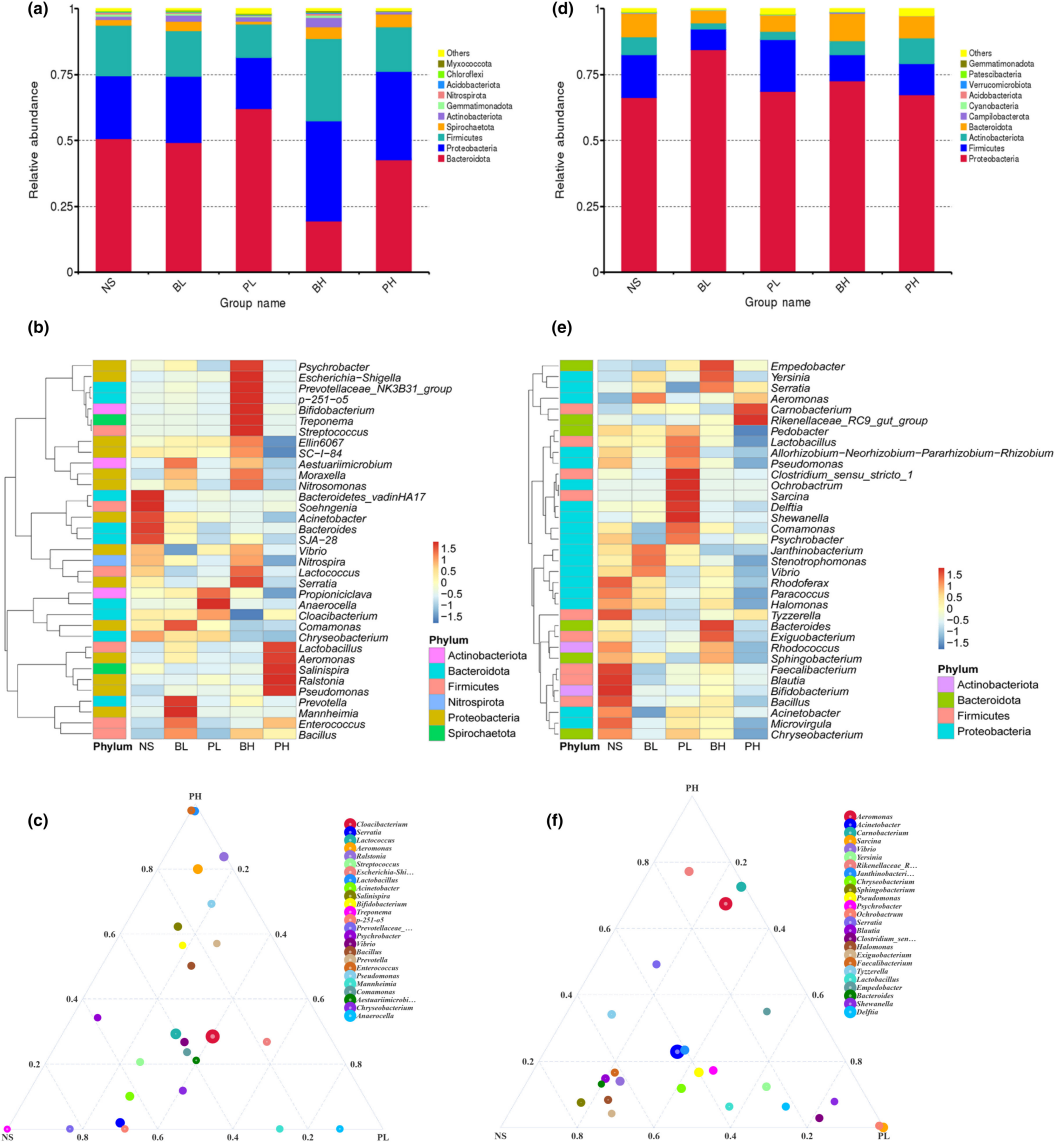
The compositions of the intestinal microbiota of *Limnodrilus hoffmeisteri* and *Propsilocerus akamusi* under different treatments. (a) phylum level of *L. hoffmeisteri*, (b) genus level of *L. hoffmeisteri*, (c) ternary plots of genus of *L. hoffmeisteri*, (d) phylum level of *P. akamusi*, (e) genus level of *P. akamusi*, (f) ternary plots of genus of *P. akamusi*. *N* = 5.

The secretion solutions of different snails have been found to influence the intestinal microbial structure of two benthic animals, with the effect of *P. canaliculata* being significantly greater than that of the native snails. Bacteroidota, Proteobacteria, and Firmicutes were the most abundant bacterial phyla in the NS group for *L. hoffmeisteri*. However, the abundance of Bacteroidota decreased in both the BH and PH groups (Figure 3a), indicating that *P. canaliculata* and the native snail secretion solutions, particularly at high concentrations, significantly influence the dominant intestinal microbiota of *L. hoffmeisteri*. Furthermore, exposure to different snail secretion solutions altered the abundance of certain genera. For instance, *Serratia*, one of the dominant bacteria in the NS group, exhibited significantly low relative abundance in the PH group.

Conversely, *Aeromonas*, one of the dominant bacteria in the PH group, had relatively low abundance in the other groups. Similarly, the relative abundance of *Pseudomonas* was highest in the PH group compared to other groups (Figure 3b). The most abundant bacterial phyla in the NS group were Proteobacteria, Firmicutes, and Bacteroidota for *P. akamusi* (Figure 3d). The relative abundance of Halomonas was higher in the NS group and extremely low in the PH group (Figure 3e). Moreover, the relative abundance of *Aeromonas* was significantly low in the NS group but became the most abundant microbiota in the PH group, consistent with the results for *L. hoffmeisteri*. The gut microbiota of *L. hoffmeisteri* in the PH group were enriched with bacteria such as *Enterococcus* and *Aeromonas* (Figure 3c), while the gut microbiota of *P. akamusi* in the PH group were also enriched in *Aeromonas* compared to the other groups (Figure 3f). *Aeromonas* became the dominant microbiota in the intestines of *L. hoffmeisteri* and *P. akamusi* after exposure to high concentrations of *P. canaliculata* secretion solution.

## 4 DISCUSSION

The success of invasive species in colonizing native ecosystems depends primarily on the stability of the native biological community (Kimbro et al., 2013). Invasive species influence microorganisms and other organisms directly or indirectly by releasing allelochemicals (Zhang et al., 2021), enabling them to monopolize resources and promote their reproductive success by eliminating other organisms through these secretions (Parras & Casadío, 2006). In the early 21st century, researchers demonstrated that *P. canaliculata* is associated with water quality deterioration and the alteration of biological community structure (Carlsson et al., 2004; Carlsson & Brönmark, 2006). Bioturbators, including *L. hoffmeisteri* and *P. akamusi*, play a crucial role in maintaining ecosystem stability and can modify the nitrogen and phosphorus content and distribution through bioturbation (Reitzel et al., 2013). Therefore, this may potentially influence the process of *P. canaliculata* invasion. However, it remains unclear whether the secretions of *P. canaliculata* affect the survival and function of bioturbators.

The detrimental effects of *P. canaliculata* secretions on two crucial bioturbators in the ecosystem, *L. hoffmeisteri* and *P. akamusi*, were investigated in the current study. Furthermore, the absence of substrate could potentially influence the behavior of *L. hoffmeisteri*, especially causing them prone to gather together. Despite the absence of substrate, *L. hoffmeisteri* stayed mostly within its nucleus in the NS group and exhibited a rapid aggregation rate, suggesting high vitality. The primary factor influencing its behavior appears to be the presence of *P. canaliculata*. The exposure of *L. hoffmeisteri* to *P. canaliculata* secretions significantly altered its migratory behavior and population identification ability. Compared to the native snail *B. aeruginosa*, the secretions of *P. canaliculata* induced a state of heightened alertness in *L. hoffmeisteri*, leading to the dispersal of numerous individual worms from the nuclear population. Furthermore, the secretions of *P. canaliculata* disrupted the mutual recognition between different populations of *L. hoffmeisteri*, hindering their integration into large population clusters. The increased individual escape and difficulty in aggregation resulted in the decline of the nucleus population of *L. hoffmeisteri*, which may have significant implications for bioturbation processes, as well as the physical structure, pore‐water chemistry, microbial activity, and nutrient cycling of the freshwater ecosystem (Anschutz et al., 2012; Florian et al., 2018; Navel et al., 2011; Nogaro & Burgin, 2014).

The impact of invasive species on native species and whether they can cause oxidative stress are poorly understood. There are few reports on whether invasive species can act as stressors and induce oxidative stress in native species. Leza et al. (2019) demonstrated that invasive bees (*Vespa velutina*) can induce oxidative stress in native honeybees. Furthermore, *P. canaliculata* secretions induce oxidative stress in the bioturbators *L. hoffmeisteri* and *P. akamusi*.

Superoxide dismutase (SOD) is the primary defense against oxidative damage (Zelko et al., 2002), while catalase (CAT) plays a significant role in the biological defense system by decomposing hydrogen peroxide (Dai et al., 2020), Glutathione (GSH) is essential for maintaining normal immune system function and has antioxidant and detoxification properties (Yang & Chen, 2011). Moreover, Malondialdehyde (MDA) is a major product of lipid peroxidation (Birben et al., 2012). In the current study, both high and low concentrations of *P. canaliculata* secretions significantly increased the SOD activity of bioturbators. Moreover, high concentrations of *P. canaliculata* secretions increased CAT activity in *L. hoffmeisteri*, while the low GSH activity in *L. hoffmeisteri* and *P. akamusi* indicates that *P. canaliculata* secretions activated the detoxification system of two bioturbators. High concentrations of *P. canaliculata* secretions induced lipid peroxidation in *L. hoffmeisteri* and *P. akamusi*, increasing the MDA concentration. These findings suggest that SOD and CAT are involved in combating the oxidative stress caused by *P. canaliculata* invasion. Chironomid larvae demonstrate notable tolerance and flexibility to environmental pollution (Ding et al., 2022). As Chironomid leave the water at the adult stage, they are not constantly exposed to the threat of *P. canaliculata* invasion throughout their lives. This suggests that *L. hoffmeisteri* may serve as a more sensitive indicator species for detecting the potential invasion of *P. canaliculata* compared to Chironomid larvae. Oxidative stress assessment and microbial community structure analysis in soil bioturbation species‐earthworms can be valuable indicators for evaluating pollutant impacts (He et al., 2021). Oxidative stress also affects the disruption of bioturbator performance, which has significant environmental implications. As bioturbators in sediments, *L. hoffmeisteri* and *P. akamusi* may participate in the removal of pollutants through their intestinal microbiota (Jang et al., 2020).

The intestinal microbiota participates in metabolism, immunity, and detoxification, which are essential for health and metabolism (Tremaroli & Backhed, 2012). Changes in the composition, quantity, or proportion of the intestinal microbiota due to disruptions in the external environment can cause dysbiosis (Olszak et al., 2012; Persico & Napolioni, 2013), which may influence the survival, reproduction, and environmental adaptation of the host (Audebert et al., 2016). The secretions of *P. canaliculata* have detrimental effects on the intestinal microbiota of *L. hoffmeisteri* and *P. akamusi*. The high concentration of *P. canaliculata* secretions can disrupt the loss of intestinal microbial diversity in *L. hoffmeisteri* and *P. akamusi*, allowing pathogenic microorganisms to colonize their intestines. *L. hoffmeisteri* is an important bioturbator in the ecosystem, especially for nitrogen and phosphorus removal, sewage treatment, and substance transformation (Hedman et al., 2011; Zhang, Gu, et al., 2010; Zhang, Hendrix, et al., 2010). The secretion‐induced changes in the intestinal microbiota caused by *P. canaliculata* decreased the abundance of microorganisms responsible for nitrogen and phosphorus pollution treatment, such as *Nitrospira*, *Nitrosomonas*, and *Halomonas*, while the abundance of these microorganisms remained unchanged or increased upon treatment with the secretions of *B. aeruginosa*. The presence of *P. canaliculata* could hinder the pollutants removing efficiency of bioturbators and exacerbating ecosystem pollution. *Enterococcus* is a common pathogen that can cause infections (Lee et al., 2020). *Enterococcus* were enriched in the gut of *L. hoffmeisteri* in the PH group. *Aeromonas* is recognized as a key and prevalent bacterium in the gut microbiome of *P. canaliculata* (Chen et al., 2021; Li et al., 2022; Lin et al., 2023). In addition, *Aeromonas* infection has been shown to affect animal behavior, induce oxidative stress, and even result in mortality (Yang et al., 2022). *Aeromonas* was found to be enriched in the gut of *L. hoffmeisteri* and *P. akamusi* of the PH group, suggesting that *Aeromonas* and other pathogens in the secretion solutions of *P. canaliculata* could be significant factors in impacting native species. These pathogens, including *Aeromonas*, *Enterococcus* are captured by bioturbators and colonize in their intestines, ultimately resulting in stress for the host. Conversely, the presence of *P. canaliculata* reduces the abundance of metabolism‐related taxa such as *Serratia* (Xu et al., 2016), suggesting that *P. canaliculata* secretions may affect the metabolism of *L. hoffmeisteri*. Intestinal microbiome disruption can increase the permeability of the intestinal barrier, resulting in the leakage of displaced bacteria and intestinal‐derived products, eventually leading to inflammation and oxidative stress (Meng et al., 2018). The decrease in the relative abundance of *Acinetobacter* is a typical indicator of oxidative stress in organisms (Zhao et al., 2021); Exposure to *P. canaliculata* secretions reduced the relative abundance of this flora in the gut of *L. hoffmeisteri*.

Changes in the intestinal microbiota of *P. akamusi* caused by *P. canaliculata* and *B. aeruginosa* secretions showed consistent patterns compared to *L. hoffmeisteri*. The increased presence of *Serratia* and *Carnobacterium* in the intestinal flora of chironomid larvae suggests that these secretions may enhance glucose metabolism. Conversely, snails reduced the abundance of *Pseudomonas*, *Rhodococcus*, and *Sphingomonas* in the intestine of chironomid larvae, with *P. canaliculata* showing a profound impact compared to *B. aeruginosa*. *P. canaliculata* can cause metabolic toxicity and eliminate the organic matter degradation ability of chironomid larvae (Yang & Chen, 2011).

The analysis of biological functions suggests that the secretions of *P. canaliculata* may negatively influence the intestinal flora immunity of *P. akamusi*. The intestinal microbiota of *L. hoffmeisteri* is more sensitive to *P. canaliculata* secretions during the invasion. Moreover, this sensitivity is amplified as the density of *P. canaliculata* invasion increases, leading to the deterioration of the intestinal microbiota.

## 5 CONCLUSION

The acute exposure to secretion solutions from *P. canaliculata* had a significant impact on the behavior of bioturbator *L. hoffmeisteri*. This caused a heightened level of alertness, leading to increased migration, as well as a decrease in the population identification ability. Furthermore, the secretions of *P. canaliculata* induced oxidative stress and lipid peroxidation in *L. hoffmeisteri* and *P. akamusi*, as well as causing changes in community structure and diversity of the intestinal microbiota of bioturbators. The abundance of nitrogen and phosphorus processing, metabolism, and detoxification‐related microbiota were severely affected following exposure to *P. canaliculata* secretion solutions. Notably, *L. hoffmeisteri* is considered to be a more sensitive bioturbator to the invasion of *P. canaliculata*. This study provides valuable insights into the impact of *P. canaliculata* invasion on native organisms and ecosystems, as well as shedding light on the underlying mechanism of stress on native species.

## AUTHOR CONTRIBUTIONS

**Mingyuan Liu:** Conceptualization (lead); data curation (lead); formal analysis (lead); methodology (lead); project administration (equal); validation (equal); visualization (lead); writing – original draft (lead). **Changrun Sui:** Data curation (equal); validation (lead); writing – original draft (equal). **Baolong Wang:** Formal analysis (equal); investigation (equal). **Ruipin Huang:** Formal analysis (equal); investigation (equal); validation (equal). **Weixiao Zhang:** Formal analysis (equal); investigation (equal). **Tao Zhang:** Formal analysis (equal); investigation (equal). **Qian Zhang:** Conceptualization (equal); data curation (equal); funding acquisition (lead); methodology (equal); project administration (lead); resources (lead); supervision (lead); validation (equal); visualization (equal); writing – review and editing (lead). **Ying Liu:** Funding acquisition (lead); methodology (equal); project administration (lead); resources (lead); validation (equal); visualization (equal); writing – review and editing (equal).

## FUNDING INFORMATION

This study was funded by the earmarked fund for China Agriculture Research System (CARS‐49), the Basic Scientific Research Project of Educational Department of Liaoning province (LJKMZ20221107), the Overseas Training Program for Innovation Team, Educational Department of Liaoning Province (201818), Basic Scientific Research Project of Educational Department of Liaoning province (2020RQ109), Joint Fund of General Research Project of Liaoning Province (2023‐MSLH‐007).

## CONFLICT OF INTEREST STATEMENT

The authors declare no conflicts of interest.

## Supporting information


Data S1

## Data Availability

The data are placed in the submission folder ‘Data S1’ for reference.
