# Supplementary material for: Effects of short‐term exposure to Pomacea canaliculata secretions on Limnodrilus hoffmeisteri and Propsilocerus akamusi: A study based on behavior, intestinal microbiota, and antioxidant system
Source: Ecol Evol. 2024 Jun 25;14(6):e11591. doi: 10.1002/ece3.11591 (PMC11199190; doi:10.1002/ece3.11591)
Supplement: Supplementary file 1 — Data S1 [file ECE3-14-e11591-s001.doc]

Fig.1 A

|  | PH | PL | BH | BL | NS |
| --- | --- | --- | --- | --- | --- |
| 0 | 11.111 | 44.444 | 11.111 | 66.667 | 88.889 |
| 1—10 | 0 | 33.333 | 0 | 33.333 | 11.111 |
| 11—50 | 0 | 22.222 | 44.444 | 0 | 0 |
| 51—100 | 55.556 | 0 | 44.444 | 0 | 0 |
| ＞100 | 33.333 | 0 | 0 | 0 | 0 |

B

0 5 10 15 20 25 30 35 40 45 50 55 60

NS 0 33.333 44.444 55.556 66.667 77.778 77.778 88.889 100 100 100 100 100

PL 0 11.111 22.222 22.222 33.333 33.333 44.444 55.556 66.667 77.778 88.889 100 100

PH 0 0 11.111 22.222 22.222 22.222 33.333 44.444 44.444 55.556 88.889 88.889 100

BL 0 0 33.333 44.444 66.667 66.667 77.778 77.778 77.778 88.889 100 100 100

BH 0 22.222 33.333 33.333 44.444 55.556 66.667 66.667 77.778 77.778 88.889 100 100

Fig.2

| Species | Group | SOD | CAT | GSH | MDA |
| --- | --- | --- | --- | --- | --- |
| *L. hoffmeister* | NS | | 22.8763 | | --- | | 16.5318 | | 19.4017 | | | 23.6682 | | --- | | 15.7762 | | 19.0136 | | | 1.1711 | | --- | | 1.4065 | | 1.4863 | | | 4.1166 | | --- | | 3.0392 | | 2.9593 | |
|  | BL | \| 17.6339 \| \| --- \| \| 16.126 \| \| 20.3257 \| | \| 21.2018 \| \| --- \| \| 21.1973 \| \| 18.2869 \| | \| 1.3279 \| \| --- \| \| 1.0472 \| \| 0.6995 \| | \| 4.6028 \| \| --- \| \| 6.4978 \| \| 3.4259 \| |
|  | BH | \| 39.9761 \| \| --- \| \| 42.4575 \| \| 40.0486 \| | \| 11.4988 \| \| --- \| \| 16.1084 \| \| 15.2391 \| | \| 0.8489 \| \| --- \| \| 0.6556 \| \| 0.6219 \| | \| 4.6648 \| \| --- \| \| 5.8247 \| \| 5.6792 \| |
|  | PL | \| 34.2165 \| \| --- \| \| 22.8757 \| \| 47.7921 \| | \| 13.6365 \| \| --- \| \| 10.7327 \| \| 15.8554 \| | \| 0.7647 \| \| --- \| \| 0.8055 \| \| 0.7789 \| | \| 6.5973 \| \| --- \| \| 6.4985 \| \| 6.9456 \| |
|  | PH | \| 39.8468 \| \| --- \| \| 40.7978 \| \| 53.3643 \| | \| 70.2362 \| \| --- \| \| 63.9494 \| \| 76.3664 \| | \| 0.9374 \| \| --- \| \| 0.7788 \| \| 0.7655 \| | \| 11.5051 \| \| --- \| \| 17.5887 \| \| 16.2324 \| |
| *P. akamusi* | NS | | 27.9112 | | --- | | 26.3578 | | 20.3715 | | | 4.8569 | | --- | | 2.05 | | 1.9402 | | | 1.7929 | | --- | | 1.3775 | | 1.1291 | | | 9.7849 | | --- | | 8.2307 | | 7.6903 | |
|  | BL | \| 22.8785 \| \| --- \| \| 17.2409 \| \| 24.0415 \| | \| 0.6001 \| \| --- \| \| 3.5864 \| \| 0.4879 \| | \| 2.0321 \| \| --- \| \| 1.2519 \| \| 1.6112 \| | \| 24.0174 \| \| --- \| \| 19.374 \| \| 23.9729 \| |
|  | BH | \| 43.2735 \| \| --- \| \| 45.9254 \| \| 47.0322 \| | \| 4.6176 \| \| --- \| \| 7.4281 \| \| 10.2892 \| | \| 0.8853 \| \| --- \| \| 1.1665 \| \| 0.8541 \| | \| 11.1053 \| \| --- \| \| 13.6044 \| \| 11.2747 \| |
|  | PL | \| 67.2214 \| \| --- \| \| 35.4993 \| \| 34.4864 \| | \| 4.6199 \| \| --- \| \| 7.2592 \| \| 1.0615 \| | \| 0.8503 \| \| --- \| \| 0.8689 \| \| 0.9632 \| | \| 15.3639 \| \| --- \| \| 10.7778 \| \| 9.0856 \| |
|  | PH | \| 71.1202 \| \| --- \| \| 50.588 \| \| 73.9759 \| | \| 5.7958 \| \| --- \| \| 4.7881 \| \| 2.0039 \| | \| 1.013 \| \| --- \| \| 0.8026 \| \| 1.0286 \| | \| 16.4118 \| \| --- \| \| 17.2971 \| \| 15.9218 \| |

Table 1

*L. Hoffmeister*

| Sample_Name | chao1 | observed_otus | shannon | simpson |
| --- | --- | --- | --- | --- |
| NS1 | 389.837 | 389 | 4.492 | 0.843 |
| NS2 | 537.872 | 532 | 5.087 | 0.889 |
| NS3 | 511.652 | 505 | 5.187 | 0.893 |
| BL1 | 590.923 | 590 | 5.369 | 0.872 |
| BL2 | 596.571 | 588 | 5.298 | 0.833 |
| BL3 | 509 | 504 | 4.826 | 0.828 |
| PL1 | 336.419 | 330 | 3.388 | 0.724 |
| PL2 | 590.571 | 582 | 6.209 | 0.941 |
| PL3 | 200.565 | 199 | 2.505 | 0.514 |
| BH1 | 416.667 | 413 | 4.766 | 0.84 |
| BH2 | 486 | 486 | 6.119 | 0.924 |
| BH3 | 500.774 | 499 | 4.918 | 0.862 |
| PH1 | 391.256 | 376 | 4.82 | 0.875 |
| PH2 | 184 | 184 | 2.432 | 0.62 |
| PH3 | 284 | 269 | 3.864 | 0.812 |

*P. akamusi*

| Sample_Name | chao1 | observed_otus | shannon | simpson |
| --- | --- | --- | --- | --- |
| NS1 | 542 | 542 | 5.784 | 0.884 |
| NS2 | 550.037 | 550 | 5.265 | 0.855 |
| NS3 | 802.964 | 801 | 7.432 | 0.978 |
| BL1 | 661.143 | 659 | 6.314 | 0.943 |
| BL2 | 279.833 | 279 | 4.079 | 0.822 |
| BL3 | 142.214 | 139 | 1.261 | 0.288 |
| PL1 | 456.353 | 456 | 5.238 | 0.869 |
| PL2 | 262.4 | 261 | 4.085 | 0.82 |
| PL3 | 709.656 | 709 | 6.946 | 0.972 |
| BH1 | 537.048 | 528 | 4.358 | 0.823 |
| BH2 | 521.5 | 521 | 5.686 | 0.905 |
| BH3 | 659.455 | 659 | 6.687 | 0.952 |
| PH1 | 210.176 | 210 | 3.592 | 0.778 |
| PH2 | 325.875 | 325 | 4.954 | 0.902 |
| PH3 | 123 | 122 | 2.61 | 0.722 |

Fig 3

*L. Hoffmeister* of phylum level

Taxonomy NS1 NS2 NS3 BL1 BL2 BL3 PL1 PL2 PL3 BH1 BH2 BH3 PH1 PH2 PH3

Bacteroidota 0.588196601 0.556404166 0.377854924 0.493586698 0.519605335 0.463000183 0.623387539 0.459492052 0.777306779 0.159693039 0.210962909 0.214215238 0.253572081 0.583190206 0.44005116

Proteobacteria 0.166636214 0.196272611 0.353955783 0.334478348 0.23486205 0.185273159 0.101479993 0.362031792 0.120190024 0.669870272 0.274218893 0.196930386 0.409574274 0.350959255 0.250721725

Firmicutes 0.190060296 0.18070528 0.199598027 0.09903161 0.148547415 0.271076192 0.220317924 0.109007857 0.05174493 0.095011876 0.38432304 0.455545405 0.22280285 0.043961264 0.238187466

Spirochaetota 0.016553992 0.023424082 0.024410744 0.043997807 0.021121871 0.045386443 0.01399598 0.00760095 0.01121871 0.025141604 0.045788416 0.064790791 0.093769413 0.00518911 0.044984469

Actinobacteriota 0.015238443 0.010634022 0.015640417 0.007198977 0.045971131 0.015896218 0.03171935 0.014909556 0.002484926 0.019513978 0.05645898 0.028357391 0.014982642 0.002704184 0.011839942

Gemmatimonadota 0.006650831 0.009245386 0.007710579 0.00445825 0.00687009 0.004750594 0.001059748 0.014288325 0 0.007089348 0.007162434 0.010341677 0.001096291 0 0.00204641

Nitrospirota 0.006066143 0.005517997 0.005262196 0.003690846 0.004787137 0.003508131 0.000548145 0.006614288 0 0.006139229 0.004933309 0.007089348 0.001279006 0 0.001096291

Acidobacteriota 0.002448383 0.003361959 0.003325416 0.00277727 0.00445825 0.003069614 0.000621232 0.006102686 0 0.004202448 0.003946647 0.005627627 0.000182715 7.31E-05 0.000694318

Chloroflexi 0.002594555 0.002923442 0.004714051 0.001315549 0.004348621 0.002156039 0.000292344 0.004129362 0 0.002375297 0.002302211 0.004019733 0.000475059 0.000109629 0.000694318

Myxococcota 0.001059748 0.001644436 0.001461721 0.00204641 0.003179244 0.001754065 0.000255801 0.004348621 0 0.003873561 0.003361959 0.004677508 0.000146172 0 0.000146172

Others 0.004494793 0.009866618 0.006066143 0.007418235 0.006248858 0.004129362 0.006321944 0.011474511 0.037054632 0.007089348 0.006541202 0.008404897 0.002119496 0.013813265 0.009537731

*L. Hoffmeister* of genus level ( Taxa summary)

Taxon NS1 NS2 NS3 BL1 BL2 BL3 PL1 PL2 PL3 BH1 BH2 BH3 PH1 PH2 PH3

Clostridium_sensu_stricto_3 0.0 0.0 0.0 33.0 26.0 108.0 0.0 231.0 0.0 20.0 46.0 43.0 0.0 0.0 0.0

Bacteroides 119.0 213.0 48.0 110.0 55.0 64.0 2.0 92.0 26.0 25.0 117.0 19.0 50.0 104.0 5.0

Rikenellaceae 60.0 45.0 73.0 90.0 52.0 33.0 6.0 238.0 0.0 142.0 70.0 31.0 1.0 0.0 0.0

Bifidobacterium 15.0 15.0 39.0 27.0 68.0 53.0 0.0 51.0 0.0 39.0 1289.0 45.0 148.0 0.0 0.0

Ellin6067 57.0 66.0 56.0 64.0 108.0 59.0 13.0 225.0 0.0 105.0 109.0 149.0 0.0 0.0 9.0

Treponema 7.0 7.0 13.0 16.0 5.0 1.0 0.0 0.0 0.0 0.0 1187.0 6.0 0.0 0.0 0.0

Prevotellaceae_UCG-003 18.0 19.0 18.0 92.0 11.0 4.0 0.0 11.0 0.0 7.0 319.0 3.0 0.0 0.0 0.0

Enterococcus 3.0 3.0 7.0 64.0 49.0 675.0 0.0 0.0 0.0 5.0 28.0 91.0 596.0 0.0 5.0

Bacteroidetes_vadinHA17 141.0 226.0 183.0 25.0 30.0 10.0 14.0 96.0 0.0 22.0 18.0 28.0 39.0 0.0 47.0

Bacillus 150.0 126.0 141.0 462.0 566.0 65.0 224.0 160.0 0.0 107.0 108.0 746.0 416.0 0.0 381.0

Ralstonia 14.0 8.0 17.0 17.0 28.0 49.0 13.0 12.0 1403.0 17.0 13.0 25.0 24.0 7532.0 20.0

Streptococcus 114.0 86.0 157.0 126.0 145.0 87.0 58.0 96.0 12.0 132.0 7467.0 197.0 107.0 9.0 22.0

Moraxella 0.0 0.0 15.0 3.0 86.0 321.0 82.0 0.0 0.0 319.0 211.0 0.0 0.0 0.0 6.0

Prevotellaceae_NK3B31_group 20.0 18.0 13.0 102.0 20.0 3.0 0.0 11.0 0.0 0.0 873.0 0.0 0.0 0.0 0.0

Lactococcus 4228.0 4029.0 2998.0 1025.0 2176.0 4307.0 5450.0 1648.0 1314.0 1328.0 676.0 10879.0 1730.0 692.0 5733.0

Prevotella 5.0 6.0 0.0 726.0 10.0 127.0 0.0 20.0 2.0 0.0 267.0 6.0 42.0 0.0 0.0

Salinispira 329.0 467.0 171.0 183.0 176.0 168.0 349.0 145.0 93.0 646.0 30.0 98.0 1748.0 23.0 787.0

Pseudomonas 25.0 17.0 58.0 86.0 87.0 45.0 34.0 143.0 8.0 62.0 47.0 137.0 30.0 585.0 29.0

Lactobacillus 3.0 10.0 18.0 15.0 30.0 1069.0 9.0 18.0 0.0 19.0 183.0 21.0 2335.0 143.0 7.0

Propioniciclava 156.0 67.0 79.0 47.0 148.0 146.0 362.0 99.0 30.0 88.0 47.0 165.0 48.0 5.0 80.0

Escherichia-Shigella 72.0 39.0 104.0 79.0 61.0 393.0 11.0 675.0 0.0 508.0 3338.0 106.0 307.0 24.0 0.0

Aeromonas 663.0 309.0 547.0 4397.0 923.0 419.0 478.0 1029.0 233.0 427.0 246.0 390.0 9122.0 324.0 3541.0

Mannheimia 3.0 0.0 5.0 515.0 0.0 480.0 21.0 0.0 0.0 0.0 45.0 0.0 0.0 0.0 0.0

p-251-o5 25.0 22.0 21.0 96.0 26.0 13.0 0.0 30.0 0.0 8.0 1128.0 12.0 0.0 0.0 0.0

SJA-28 272.0 350.0 297.0 97.0 203.0 111.0 25.0 88.0 0.0 133.0 114.0 160.0 38.0 0.0 67.0

Comamonas 142.0 499.0 115.0 168.0 502.0 415.0 110.0 360.0 200.0 156.0 113.0 138.0 118.0 296.0 30.0

Acinetobacter 282.0 1884.0 1416.0 397.0 1214.0 432.0 302.0 980.0 308.0 426.0 555.0 313.0 132.0 50.0 396.0

SC-I-84 120.0 131.0 128.0 96.0 145.0 118.0 23.0 291.0 0.0 176.0 147.0 214.0 14.0 0.0 8.0

Cloacibacterium 14302.0 13109.0 8544.0 10790.0 12582.0 11657.0 16417.0 9834.0 20548.0 2921.0 1425.0 4773.0 6451.0 15487.0 11092.0

Serratia 813.0 224.0 4745.0 396.0 320.0 295.0 212.0 2146.0 89.0 12295.0 543.0 321.0 39.0 4.0 119.0

Anaerocella 20.0 14.0 15.0 36.0 21.0 9.0 0.0 379.0 0.0 11.0 13.0 13.0 0.0 0.0 0.0

Chryseobacterium 148.0 461.0 172.0 157.0 299.0 174.0 86.0 310.0 278.0 68.0 80.0 79.0 42.0 115.0 43.0

Vibrio 726.0 227.0 277.0 156.0 150.0 296.0 752.0 208.0 89.0 382.0 139.0 738.0 406.0 99.0 324.0

Psychrobacter 0.0 0.0 221.0 6.0 281.0 98.0 26.0 0.0 0.0 798.0 97.0 0.0 0.0 20.0 107.0

Aestuariimicrobium 74.0 72.0 52.0 48.0 493.0 73.0 82.0 99.0 21.0 74.0 48.0 377.0 47.0 4.0 56.0

1. *Akamusi* of phylum level

Taxonomy NS1 NS2 NS3 BL1 BL2 BL3 PL1 PL2 PL3 BH1 BH2 BH3 PH1 PH2 PH3

Proteobacteria 0.74691112 0.777202073 0.467995217 0.721881228 0.900677561 0.91327222 0.730437093 0.885877508 0.439511093 0.878942474 0.720791816 0.578211771 0.859891059 0.432735486 0.725282317

Firmicutes 0.07043975 0.086541783 0.326610868 0.088428325 0.061219609 0.082449847 0.117948718 0.009857845 0.464434702 0.054815996 0.103786369 0.141543776 0.01339179 0.087179487 0.256224259

Actinobacteriota 0.056011691 0.035073735 0.111491962 0.060130198 0.006244188 0.002843098 0.053992294 0.02128338 0.021469377 0.036296001 0.033426332 0.089066029 0.01155839 0.272565431 0.0099907

Bacteroidota 0.109685134 0.087976618 0.072379434 0.115876179 0.030344095 0.001089411 0.034250033 0.082290421 0.065683539 0.025348745 0.106656038 0.176511226 0.085983792 0.155626412 0.007732164

Campilobacterota 0.000743988 0.000823701 0.001913113 0.000425136 5.31E-05 5.31E-05 0.000318852 0.000106284 0.000106284 0.000956556 0.008077587 0.002949382 5.31E-05 0.000185997 0.000239139

Cyanobacteria 0.000371994 0.004835924 0.000956556 0.000823701 0.001169124 0 0.004145078 0.000345423 0.001487977 0.000212568 0.000664275 0.002205394 0.000478278 0.000185997 0.000185997

Acidobacteriota 0.000132855 0 0 0 0 0 0.001886542 0 0 0 5.31E-05 0.000132855 0 0 0

Verrucomicrobiota 0.001700545 0.000398565 0.000425136 0.001647403 0 0 0.00053142 0 0.000106284 0 0.000717417 0.000425136 0 0 0

Patescibacteria 0.000398565 0.000478278 0.001328551 0.000664275 0 0 0.001089411 0 0.000664275 0.000611133 0.000425136 0.000664275 0 0 0

Gemmatimonadota 0 0 7.97E-05 0 0 0 0.00130198 0 0.000106284 0 0 0 0 0 0

Others 0.013604358 0.006669324 0.01681945 0.010123555 0.000292281 0.000292281 0.054098578 0.000239139 0.006430185 0.002816527 0.025401887 0.008290155 0.02864355 0.05152119 0.000345423

1. *Akamusi* of genus level ( Taxa summary)

Taxon NS1 NS2 NS3 BL1 BL2 BL3 PL1 PL2 PL3 BH1 BH2 BH3 PH1 PH2 PH3

Terrisporobacter 0.0 7.0 19.0 19.0 0.0 0.0 0.0 0.0 629.0 0.0 9.0 13.0 0.0 0.0 0.0

Empedobacter 85.0 26.0 43.0 72.0 8.0 0.0 273.0 199.0 143.0 193.0 237.0 771.0 152.0 214.0 46.0

Paracoccus 202.0 171.0 317.0 499.0 4.0 17.0 84.0 32.0 187.0 78.0 162.0 212.0 31.0 81.0 11.0

Bacteroides 75.0 265.0 158.0 235.0 3.0 0.0 22.0 9.0 116.0 33.0 742.0 219.0 48.0 46.0 0.0

Faecalibacterium 36.0 99.0 902.0 94.0 5.0 6.0 35.0 25.0 302.0 31.0 291.0 210.0 24.0 250.0 5.0

Clostridium_sensu_stricto_1 16.0 59.0 148.0 40.0 4.0 4.0 5.0 0.0 1173.0 35.0 50.0 40.0 10.0 31.0 0.0

Pedobacter 301.0 195.0 182.0 550.0 31.0 0.0 55.0 100.0 594.0 18.0 139.0 353.0 43.0 47.0 61.0

Bacillus 96.0 181.0 643.0 195.0 5.0 12.0 256.0 4.0 56.0 0.0 113.0 379.0 0.0 51.0 1.0

Rhodoferax 633.0 309.0 182.0 559.0 131.0 13.0 31.0 24.0 120.0 29.0 154.0 299.0 36.0 22.0 4.0

Allorhizobium-Neorhizobium-Pararhizobium-Rhizobium 381.0 78.0 201.0 387.0 24.0 13.0 134.0 197.0 512.0 58.0 84.0 113.0 32.0 115.0 35.0

Delftia 246.0 123.0 105.0 179.0 327.0 11.0 180.0 690.0 650.0 51.0 61.0 179.0 60.0 49.0 26.0

Carnobacterium 20.0 29.0 72.0 48.0 1217.0 2883.0 3393.0 68.0 95.0 171.0 33.0 48.0 57.0 137.0 9523.0

Janthinobacterium 959.0 552.0 1105.0 3065.0 103.0 54.0 384.0 830.0 1160.0 177.0 446.0 844.0 180.0 1193.0 146.0

Pseudomonas 828.0 990.0 1337.0 1783.0 175.0 71.0 936.0 1521.0 1002.0 442.0 503.0 1316.0 469.0 760.0 83.0

Ochrobactrum 13.0 0.0 0.0 15.0 0.0 0.0 0.0 0.0 1485.0 0.0 0.0 8.0 0.0 9.0 0.0

Brevundimonas 156.0 115.0 193.0 509.0 19.0 16.0 150.0 73.0 266.0 24.0 76.0 172.0 23.0 37.0 11.0

Lactobacillus 101.0 269.0 261.0 358.0 246.0 14.0 54.0 49.0 863.0 69.0 205.0 251.0 23.0 60.0 24.0

Tyzzerella 565.0 121.0 868.0 241.0 75.0 11.0 162.0 55.0 129.0 89.0 292.0 305.0 72.0 887.0 28.0

Exiguobacterium 98.0 124.0 408.0 117.0 1.0 16.0 136.0 1.0 107.0 7.0 85.0 910.0 8.0 31.0 0.0

Aeromonas 618.0 3379.0 930.0 1283.0 20451.0 32450.0 14327.0 1475.0 816.0 21690.0 2122.0 521.0 25914.0 956.0 17725.0

Stenotrophomonas 120.0 147.0 171.0 108.0 583.0 11.0 190.0 46.0 180.0 18.0 93.0 153.0 17.0 11.0 0.0

Yersinia 191.0 214.0 209.0 184.0 4454.0 283.0 1162.0 359.0 79.0 4140.0 3431.0 244.0 139.0 89.0 84.0

Rikenellaceae_RC9_gut_group 224.0 156.0 499.0 35.0 40.0 4.0 335.0 315.0 117.0 95.0 74.0 1440.0 1508.0 4067.0 26.0

Legionella 591.0 8.0 12.0 5.0 0.0 0.0 0.0 0.0 6.0 3.0 2.0 9.0 0.0 0.0 0.0

Blautia 64.0 254.0 1320.0 115.0 16.0 12.0 12.0 14.0 483.0 30.0 408.0 200.0 60.0 311.0 1.0

Acinetobacter 17668.0 18049.0 6478.0 8481.0 3841.0 531.0 3772.0 25241.0 5638.0 4348.0 14530.0 12962.0 3227.0 10864.0 8715.0

Shewanella 34.0 23.0 27.0 43.0 156.0 5.0 711.0 29.0 28.0 43.0 23.0 29.0 26.0 29.0 19.0

Halomonas 332.0 394.0 551.0 1032.0 11.0 40.0 139.0 83.0 228.0 184.0 410.0 404.0 36.0 124.0 0.0

Rhodococcus 269.0 257.0 402.0 445.0 48.0 22.0 201.0 304.0 114.0 96.0 493.0 286.0 73.0 277.0 45.0

Serratia 428.0 291.0 134.0 182.0 663.0 338.0 308.0 57.0 36.0 59.0 1476.0 136.0 1159.0 37.0 20.0

Sphingobacterium 1299.0 880.0 978.0 1710.0 547.0 22.0 100.0 163.0 462.0 74.0 1125.0 2020.0 116.0 170.0 30.0

Chryseobacterium 1724.0 1296.0 503.0 1300.0 459.0 15.0 310.0 2252.0 558.0 294.0 1143.0 992.0 223.0 579.0 93.0

Sarcina 0.0 9.0 0.0 0.0 0.0 4.0 0.0 0.0 8909.0 0.0 4.0 2.0 0.0 0.0 0.0

Psychrobacter 133.0 576.0 561.0 10.0 3.0 0.0 1488.0 73.0 109.0 61.0 16.0 283.0 307.0 281.0 30.0

Vibrio 902.0 1358.0 1837.0 5068.0 28.0 195.0 449.0 306.0 856.0 523.0 1460.0 1404.0 189.0 678.0 67.0
